# Temporal Associations of Daily Changes in Sleep and Depression Core Symptoms in Patients Suffering From Major Depressive Disorder: Idiographic Time-Series Analysis

**DOI:** 10.2196/17071

**Published:** 2020-04-23

**Authors:** Noah Lorenz, Christian Sander, Galina Ivanova, Ulrich Hegerl

**Affiliations:** 1 Department of Psychiatry and Psychotherapy Faculty of Medicine Leipzig University Leipzig Germany; 2 Research Centre of the German Depression Foundation Leipzig Germany; 3 Institute for Applied Informatics Leipzig Germany; 4 Department of Psychiatry, Psychosomatics and Psychotherapy Goethe-Universität Frankfurt Frankfurt Germany

**Keywords:** depression, sleep, time series, idiographic, self-management

## Abstract

**Background:**

There is a strong link between sleep and major depression; however, the causal relationship remains unclear. In particular, it is unknown whether changes in depression core symptoms precede or follow changes in sleep, and whether a longer or shorter sleep duration is related to improvements of depression core symptoms.

**Objective:**

The aim of this study was to investigate temporal associations between sleep and depression in patients suffering from major depressive disorder using an idiographic research approach.

**Methods:**

Time-series data of daily sleep assessments (time in bed and total sleep time) and self-rated depression core symptoms for an average of 173 days per patient were analyzed in 22 patients diagnosed with recurrent major depressive disorder using a vector autoregression model. Granger causality tests were conducted to test for possible causality. Impulse response analysis and forecast error variance decomposition were performed to quantify the temporal mutual impact of sleep and depression.

**Results:**

Overall, 11 positive and 5 negative associations were identified between time in bed/total sleep time and depression core symptoms. Granger analysis showed that time in bed/total sleep time caused depression core symptoms in 9 associations, whereas this temporal order was reversed for the other 7 associations. Most of the variance (10%) concerning depression core symptoms could be explained by time in bed. Changes in sleep or depressive symptoms of 1 SD had the greatest impact on the other variable in the following 2 to 4 days.

**Conclusions:**

Longer rather than shorter bedtimes were associated with more depression core symptoms. However, the temporal orders of the associations were heterogeneous.

## Introduction

Most patients with depression suffer from sleep disturbances, which mainly occur in the form of difficulty in initiating or maintaining sleep. Sleep disturbances have often been reported by patients and clinicians as an early sign or symptom at depression onset. This assumption has been supported by studies showing that insomnia and short sleep duration increase the risk of depression onset [1,2]. In addition, sleep disturbances are a risk factor for recurrent depression [3] and worsening symptoms of depression [4] (also see [5] or [6] for an overview). These studies indicate that sleep plays a causal role in the development of depression. This interpretation was supported by findings of studies focusing on the treatment of insomnia with cognitive behavior therapy, which has been shown to substantially reduce depression symptoms [7,8]. One study also showed that cognitive behavior therapy focused on treating insomnia symptoms was equally effective in reducing depression core symptoms as therapy focused on treating depression symptoms [9].

However, the association between sleep disturbances and depression can also be explained by a common pathogenic factor that causes both symptoms. The arousal regulation model of affective disorders describes upregulated brain arousal as such a central pathogenic factor [10], which is commonly found in patients with major depressive disorder. The withdrawal and sensation avoidance observed in major depressive disorder is considered to be an autoregulatory reaction of the individual in response to the high brain arousal. Upregulated brain arousal can also provide an explanation for other coexisting symptoms such as insomnia or anxiety. According to this concept, arousal regulation can be influenced by factors such as increasing sleep pressure via sleep deprivation, which counteracts the upregulated brain arousal, whereas extended sleep durations maintain the pathogenic factor (arousal). These assumptions were supported by an experimental study revealing that an increase in time in bed and total sleep time has negative effects on mood [11]. Even partial sleep deprivation of 2 hours was shown to have an antidepressive effect in some patients [12]. Moreover, one of the decisive interventions applied in cognitive behavioral therapy for insomnia is sleep restriction, in which patients experience a significant reduction in bedtime. Thus, consistent with sleep restriction studies in the context of depression, it is possible that this reduction in time in bed leads to an improvement in depression symptoms through the mechanism described by the arousal model. This association has already been convincingly demonstrated for patients with bipolar disorder in a time-series study design [13]. The authors investigated the temporal association between sleep and mood over the course of an average of 169 days, finding that a decrease in sleep duration was followed by an increase in mood the following day for 34% of the patients, and the same association was found between bedrest (awake in bed) and mood for 22% of the patients, with no positive cross-correlations identified for sleep duration. However, this is one of only few studies that have investigated the relationship between sleep and mood in an idiographic research design.

Idiographic research is based on within-subject analysis techniques. Instead of analyzing cohorts to explain variance in the population, the aim of an idiographic approach is to explain variance within individuals without seeking to generalize the results to other individuals. Such analyses require repeated measurements for the same individual. The experience sampling method or momentary ecological assessment is a promising research technique to collect repeated measures within individuals for an idiographic analysis [14]. One of the main advantages of idiographic research is that it is a more person-centered approach, since patterns within individuals can be investigated over time. For example, this method allows for investigating temporal associations with various time lags between several variables for a given individual, enabling identifying indications for cause-effect relationships of several variables within an individual [15]. Therefore, this approach can provide results with high clinical relevance, as they apply to specific individuals in specific contexts. In particular, if interindividual differences between patients are to be assumed, an idiographic research design can avoid the disadvantages of group statistics that risk blurring the relevant effects on the individual level. Molenaar et al [16] provide a more detailed discussion and comparison of interindividual and intraindividual research methodologies.

For both clinicians and individual patients, it would be highly relevant to understand how changes in sleep are related to changes in depressive symptoms. For example, if shorter bedtimes lead to a reduction in depressive symptoms in individual patients, this information can be used by both the clinician for treatment and by the patient for self-management. Idiographic time-series studies are useful to explore these questions, and some authors have shown that ecological momentary assessment in combination with time-series analysis are useful tools for this purpose [17,18].

The goal of the present study was to investigate the relationship between sleep and depression core symptoms by applying selected time-series methods on long-term collected sleep and self-rating data. We pursued three explorative aims. The first aim was to investigate the number of patients demonstrating significant temporal associations (indicating possible causal relationships) between time in bed/total sleep time and core depression symptoms. The second aim was to describe the nature of the relationship from two aspects: whether changes in sleep cause changes in depression or vice versa, and whether more or less sleep causes a reduction in depression. Finally, we sought to analyze the impact and temporal dynamics of the effects in patients with significant temporal associations by calculating the amount of variance explained in the time series and by analyzing how changes of 1 SD in a time series affect the other time series over a 10-day period.

## Methods

### Sensor-Based System for Therapy Support and Management of Depression Research Project

The data analyzed in this study were obtained from the research project Sensor-based System for Therapy Support and Management of Depression (STEADY), with the broad aim of developing a digital solution allowing patients to collect data on the course of their disease using smartphones and mobile sensors. To support patients in their self-management, one aim of STEADY was to identify patient-specific patterns and associations in the recorded self-ratings and digital behavior markers that can serve as a basis for patient-specific self-management. A feasibility study was carried out within the STEADY project, which was divided into three distinct study phases targeted at investigating specific modules or features of the comprehensive STEADY system. Before and after each study phase, data were collected using printed self-assessment questionnaires, including the Pittsburg Sleep Quality Index (PSQI) [19]. During the study phase, patients were supported by weekly and later monthly telephone calls. Furthermore, a monthly on-site appointment was held at the research center of the German Depression Foundation in Leipzig, Germany.

### Participants

Participants of the STEADY feasibility study were primarily recruited at the Department of Psychiatry and Psychotherapy of the University of Leipzig, Germany. In addition, interested individuals could register in the study via a project-specific website. The study was approved by the local ethics committee of the University of Leipzig. Once written informed consent was obtained, participants were invited for a diagnostic interview to check for eligibility according to the following criteria. Inclusion screening criteria were at least 18 years of age, diagnosis of a recurrent depressive disorder, current depressive symptomatology reflected by a minimum of 14 points on the Inventory of Depressive Symptomatology Clinician (IDS-C) scale [20], undergoing professional treatment with regard to the diagnosis, and living in close proximity to the study center to facilitate regular study appointments. Exclusion criteria were psychiatric comorbidities (alcohol/drug addiction, schizophrenia, schizotypal and delusional disorders, borderline personality disorder), severe somatic disorders, acute suicidal behavior, pregnancy/lactation, and electronic implants.

Inclusion and exclusion criteria were checked using semistructured interviews on sociodemographic factors and medical history. Psychiatric diagnoses were established using the Structured Clinical Interview for Diagnostic and Statistical Manual of Depressive Disorders IV [21]. Depression severity was rated on the IDS-C scale [20] by trained raters. A total of 25 participants were included in the study. To be included in this current analysis, participants had to collect data over a continuous period of at least 130 days. Additionally, subjects were excluded if missing values exceeded 30% during this period. After applying these additional criteria, 22 of 25 participants were included in the present analysis.

### App-Based Self-Rating and Measures

According to the goals of the project, an app was developed by the STEADY consortium to support the self-rating process. The study participants performed morning and evening self-ratings using the app. The smartphone app was programmed so that morning logs were available daily from 3 am to 3 pm and evening logs were available daily from 3 pm to 3 am. Patients were asked to fill in the morning logs directly after awakening and to fill in the evening logs right before going to bed.

Two main self-ratings were considered: daily ratings of depression core symptoms and night sleep. The former considered core symptoms addressing interest and pleasure as well as feelings of depression and hopelessness such as those used in the Two-item Health Questionnaire (PHQ-2) [22] to measure depression symptoms. In the evening log, patients rated how often they experienced the symptoms during the day on a visual analogue scale from 0 (“never”) to 10 (“all the time”). A mean of the two items was calculated and used as a measure of depression core symptoms severity, hereinafter referred to as depression core symptoms. For night sleep measurement, participants were asked to fill in a sleep diary that was integrated in the morning log consisting of “go to bed time,” “get up time,” and “time spent sleeping.” Time in bed and total sleep time were then calculated from the sleep diary as the main parameters for analysis.

### Procedure

Patients were invited to the study center where they were given a general introduction to the app and the integrated morning and evening logs. Patients were instructed on how and when to fill in the logs. Monthly visits at the study center took place during the data collection period, which involved a data plausibility check (including a check for missing values), along with the opportunity to address patient questions and difficulties. Study assistants monitored the data collection process throughout this period. This involved a continuous check for missing values. When a missing value was noted, it was addressed in a phone call with the patient to obtain feedback on the reasons for missing values as a measure to reduce the occurrence of missing values.

### Data Analysis

The vector autoregression (VAR) technique was applied to analyze the collected datasets [23]. The VAR technique was originally developed for research in economic sciences, but has also been used in other fields such as neuroimaging, sociology, and meteorology. A useful advantage of the VAR technique is that it allows investigating the temporal dynamics between several time series without the need of presumptions about the possible associations. In a VAR model, all of the endogenous variables are regressed onto their own time-lagged values and the time-lagged values of all other endogenous variables. An endogenous variable is a variable that can be both a determinant and an outcome. Therefore, a VAR model enables drawing conclusions about the temporal sequence of the effects to derive indications for cause-effect relationships [23].

Several steps are required when conducting a VAR analysis. One important step is to make a decision about the maximum lag. The lag length refers to the maximum number of previous observations (eg, the previous 2 days), which is then used to estimate the current observation. Lag length selection criteria can be used to determine the optimal number of lags.

In this study, we established two models with two variables each. Each model consisted of two endogenous variables: total sleep time and depression core symptoms (model 1), and time in bed and depression core symptoms (model 2). The corresponding regression equations are listed below.

Equations for model 1:


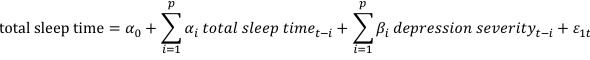



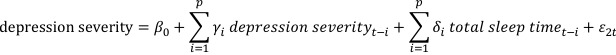


Equations for model 2:


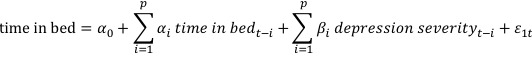



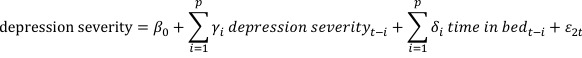


In these models, *α_i_, βi, γi*, and *δi* are coefficients to be estimated; *ε_1_* and *ε_2_* are the error terms; and *p* is the number of lags considered in the model. A maximal lag length of 7 days was used. The Akaike information criterion value was used to identify the optimal number of lags for the models. To account for trends and to obtain stationarity, the time series were passed through a linear filter using the autoregressive integrated moving average methodology [24]. To test for potential cause-effect associations, Granger causality tests were conducted. This test examines whether the past values of a variable are useful to predict another variable. A variable *x* is considered to be causally related to a variable *y* if past values of *y* and *x* predict *y* significantly better than the past values of *y* alone. The principle behind the Granger test is that a cause cannot come after an effect [15]. Therefore, testing the temporal order of associations provides information on potential causal effects. To determine the impact of each endogenous variable over time, impulse response functions (IRFs) were calculated for all patients with significant Granger causality test results. An impulse response is the temporal reaction of a dynamic system to a change of a variable (eg, change of total sleep time in our analysis). Such a change is called “shock” and is normally defined as 1 SD in the time series; this definition was adopted in the present study as it has been shown to be a reliable definition of change in clinical research [18]. Furthermore, forecast error variance decomposition (FEVD) was performed to estimate the amount of variance in a variable that can be explained by another variable in a defined time period. The R package *vars* was used for statistical analysis [25].

### Missing Values

Missing values occurred occasionally in the time series whenever a patient forgot to fill in the morning or evening logs. Since the applied analysis method cannot handle missing data, imputation methods were used to estimate missing values. In a given time series, missing values are estimated on the basis of the respective time series itself to avoid sham correlations between different time series. For this purpose, the Kalman smoothing imputation method was applied using the R package *imputeTS* [26].

## Results

### Demographics and Clinical Characteristics

Data of 22 participants (15 women, 7 men) were analyzed in this study. On average, data for 173 days (range 143-205) per participant were available. Participants were aged between 21 and 67 years (median 43.5) and had a mean IDS-C baseline score of 27.27 (SD 8.5), corresponding to a moderate severity of depression, and a mean PSQI baseline score of 8.95 (SD 4.62). Nineteen of the 22 participants (86%) were taking antidepressant medication and had already undergone psychotherapy or were in psychotherapy at the time of the study. Table 1 summarizes all demographic and clinical characteristics at the individual patient level.

**Table 1:** Demographic and clinical characteristics.

Patient	Data collection period (days)	Sex	Age (years)	Employment status	Other psychiatric disorders	PSQI^a^		IDS-C^b^	
						T1^c^	T2^d^	T1	T2
1	201	F	41	Housewife	Anxiety disorder/ agoraphobia	—^e^	11	24	14
2	150	F	50	Full time	Generalized anxiety disorder, May 2017-present	12	13	23	30
3	174	M	54	Incapacity for work due to illness	Generalized anxiety disorder	4	6	39	21
4	179	F	33	Part time	None	5	9	17	18
5	172	F	43	Full time	None	5	4	6	7
6	205	F	60	Partial retirement	None	10	9	34	34
7	199	F	44	Self-employed/ freelance	None	4	6	7	16
8	150	F	45	Full time	Pain disorder, 2010-present	18	14	9	27
9	188	F	38	Full time	None	10	18	18	38
10	156	F	33	Full time	None	7	7	11	10
11	203	M	42	Self-employed/ freelance	None	2	3	19	9
12	170	M	21	Student	None	10	10	11	15
13	154	M	53	Partial retirement	None	22	20	44	44
14	160	F	48	Self-employed/ freelance	None	7	5	15	6
15	143	M	50	Privateer	None	7	7	31	36
16	150	F	35	Sick leave	None	3	7	22	25
17	199	F	32	Full time	None	9	–	23	
18	179	F	53	Part time	Trichotillomania	8	5	15	17
19	178	F	27	Student	Attention deficit and hyperactivity disorder	–	15	20	16
20	173	F	67	Pension/early retirement	None	10	12	29	20
21	178	M	26	Part time	None	7	9	5	14
22	151	M	62	Incapacity for work due to illness	None	16	17	45	39

^a^PSQI: Pittsburg Sleep Quality Index score.

^b^IDS-C: Inventory of Depressive Symptomatology Clinician Rating.

^c^T1: before the study phase.

^d^T2: after the study phase.

^e^—: data not available.

### Granger Causality Results

**Figure 1:**
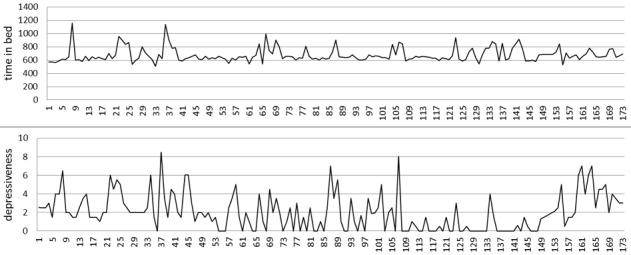
Raw time-series data of patient 2.

Granger causality tests were performed to investigate the temporal order of associations and to test for potential causal effects. In total, 11 patients showed significant Granger causal associations at a significance level of 5%. For 3 patients, time in bed Granger caused depression core symptoms (positive association), meaning that more time in bed was followed by more severe depression. In 3 patients, this association had a reversed temporal order, meaning that more depression core symptoms were followed by more time in bed. In one patient, depression core symptoms Granger caused time in bed (negative association), meaning that more depression core symptoms were followed by less time in bed. In 3 patients, total sleep time Granger caused depression core symptoms (positive association), meaning that more total sleep time was followed by more depression, whereas this temporal order was reversed in 2 patients (ie, more depression core symptoms were followed by greater total sleep time). In 3 patients, total sleep time Granger caused depression core symptoms (negative association), meaning that more total sleep time was followed by less depression core symptoms, and this temporal order was reversed for 1 patient. Figure 1 shows the raw time series (time in bed and depression core symptoms) of patient 2 as an example. Table 2 summarizes all of the statistically significant Granger causality test results.

**Table 2:** Granger causality test results.

Causality test		Positive association (n)		Negative association (n)				Total (n)
**Time in bed (TIB)**								
	TIB Granger causes depression core symptoms		3		0		3	
	Depression core symptoms Granger causes TIB		3		1		4	
	Mutual causality		0		0		0	
**Total sleep time (TST)**								
	TST Granger causes depression core symptoms		3		3		6	
	Depression core symptoms Granger causes TST		2		1		3	
	Mutual causality		0		0		0	

### Impulse Response Function

**Figure 2:**
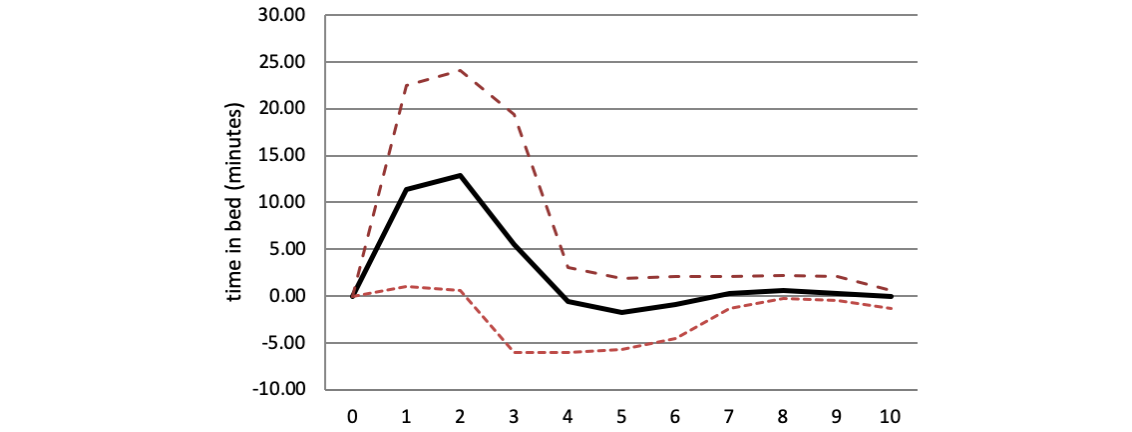
Impulse response function for patient 2. Impulse represents depression severity, response is time in bed, time horizon = 10 days; the dashed line indicates the 95% CI.

IRFs were calculated to analyze the impact of a change in the two sleep variables on depression core symptoms and vice versa over time. Figure 2 provides an example of the IRF for patient 2, showing the impact of a change (1 SD) in depression core symptoms on time in bed during a 10-day period. 

In this case, a change in depression core symptoms led to a significant increase in time in bed the following 2 days. At day 2, the effect reached its peak with an increase of 12.86 minutes in bed. At day 3, the impact was no longer significant based on a confidence interval exceeding 0. IRF coefficients can be cumulated to calculate the total impact of a change (1 SD) in a variable over time. For patient 2, a change in depression core symptoms of 1 SD led to a total increase in time in bed of 27.64 minutes during a 10-day period. Table 3 summarizes the cumulated IRF results for all patients with significant Granger causality test results.

**Table 3:** Cumulative results^a^ of impulse response analysis for all patients with significant Granger causality test results.

Granger causality		lag^b^ 0	lag 1	lag 2	lag 3	lag 4	lag 5	lag 6	lag 7	lag 8	lag 9	lag 10
**TIB^c^ Granger causes depression**												
	Patient 3	0.06	0.29	0.51	0.53	0.53	0.53	0.52	0.52	0.52	0.52	0.52
	Patient 6	0.02	0.25	0.31	0.32	0.32	0.31	0.31	0.31	0.31	0.31	0.31
	Patient 20	0.44	0.89	1.00	1.02	1.03	1.03	1.03	1.03	1.03	1.03	1.03
	Mean	0.17	0.47	0.60	0.62	0.63	0.62	0.62	0.62	0.62	0.62	0.62
**Depression Granger causes TIB**												
	Patient 10	0.00	7.46	9.17	9.35	9.34	9.33	9.33	9.33	9.33	9.33	9.33
	Patient 11	0.00	8.42	9.44	9.72	9.78	9.79	9.79	9.79	9.79	9.79	9.79
	Patient 14	0.00	12.74	13.33	13.55	13.57	13.57	13.57	13.57	13.57	13.57	13.57
	Patient 19	0.00	–7.16	–20.71	–22.59	–23.64	–23.81	–23.66	–23.63	–23.60	–23.60	–23.60
	Mean	0.00	8.95	13.16	13.80	14.08	14.13	14.09	14.08	14.07	14.07	14.07
**TST^d^ Granger causes depression**												
	Patient 6	0.06	0.27	0.32	0.32	0.32	0.32	0.32	0.32	0.32	0.32	0.32
	Patient 7	0.09	–0.16	–0.13	–0.14	–0.14	–0.14	–0.14	–0.14	–0.14	–0.14	–0.14
	Patient 14	0.08	–0.11	–0.10	–0.10	–0.10	–0.10	–0.10	–0.10	–0.10	–0.10	–0.10
	Patient 19	–0.17	–0.46	–0.48	–0.49	–0.49	–0.49	–0.49	–0.49	–0.49	–0.49	–0.49
	Patient 20	–0.24	0.20	0.23	0.23	0.23	0.23	0.23	0.23	0.23	0.23	0.23
	Patient 21	0.36	0.09	–0.03	–0.10	–0.25	–0.40	–0.40	–0.35	–0.30	–0.27	–0.24
	Mean	0.17	0.22	0.22	0.23	0.26	0.28	0.28	0.27	0.26	0.26	0.25
**Depression Granger causes TST**												
	Patient 2	0.00	9.28	20.88	27.44	28.44	27.79	27.23	27.49	28.03	28.44	28.55
	Patient 11	0.00	9.11	10.24	10.57	10.63	10.64	10.65	10.65	10.65	10.65	10.65
	Patient 22	0.00	–9.46	–9.36	–9.37	–9.37	–9.37	–9.37	–9.37	–9.37	–9.37	–9.37
	Mean	0.00	9.28	13.49	15.79	16.15	15.93	15.75	15.84	16.02	16.15	16.19

^a^Values are expressed in minutes (TIB and TST) or units on the depression scale (depression).

^b^Lag numbers correspond to days.

^c^TIB: time in bed.

^d^TST: total sleep time.

### Variance Decomposition

Table 4 summarizes the results of the FEVD analysis over a period of 10 days for all patients with significant Granger causality test results. As an example, in patient 20, 6.4% of the variance in depression core symptoms at lag 1 (the day following night sleep) could be explained by time in bed. The following day (lag 2), the percentage increased to 12.4%. At day 3 (lag 3), a peak was reached with 12.7% of the variance explained. The mean FEVD results were calculated for each temporal association across patients with respective significant Granger causal associations. On average, 10% and 6% of the variance in depression core symptoms could be explained by time in bed and total sleep time, respectively, and 3% of variance in time in bed and in total sleep time could be explained by depression core symptoms.

**Table 4:** Explained variance in depression core symptoms and time in bed at different time lags for all patients with significant Granger causality test results.

Granger causality			lag^a^ 1		lag 2		lag 3		lag 4		lag 5		lag 6		lag 8		lag 9		lag 10
**TIB^b^ Granger causes depression**																			
	Patient 3	0.00		0.07		0.12		0.12		0.12		0.12		0.12		0.12		0.12	
	Patient 6	0.00		0.04		0.05		0.05		0.05		0.05		0.05		0.05		0.05	
	Patient 20	0.06		0.12		0.13		0.13		0.13		0.13		0.13		0.13		0.13	
	Mean	0.02		0.08		0.10		0.10		0.10		0.10		0.10		0.10		0.10	
**Depression Granger causes TIB**																			
	Patient 10	0.03		0.03		0.03		0.03		0.03		0.03		0.03		0.03		0.03	
	Patient 11	0.02		0.02		0.02		0.02		0.02		0.02		0.02		0.02		0.02	
	Patient 14	0.03		0.03		0.03		0.03		0.03		0.03		0.03		0.03		0.03	
	Patient 19	0.00		0.01		0.04		0.04		0.04		0.04		0.04		0.04		0.04	
	Mean	0.02		0.02		0.03		0.03		0.03		0.03		0.03		0.03		0.03	
**TST^c^ Granger causes depression**																			
	Patient 6	0.00		0.04		0.04		0.04		0.04		0.04		0.04		0.04		0.04	
	Patient 7	0.00		0.02		0.02		0.02		0.02		0.02		0.02		0.02		0.02	
	Patient 14	0.01		0.03		0.03		0.03		0.03		0.03		0.03		0.03		0.03	
	Patient 19	0.01		0.04		0.04		0.04		0.04		0.04		0.04		0.04		0.04	
	Patient 20	0.02		0.08		0.08		0.08		0.08		0.08		0.08		0.08		0.08	
	Patient 21	0.09		0.13		0.14		0.14		0.15		0.16		0.16		0.16		0.16	
	Mean	0.02		0.06		0.06		0.06		0.06		0.06		0.06		0.06		0.06	
**Depression Granger causes TST**																			
	Patient 2	0.00		0.02		0.04		0.05		0.05		0.05		0.05		0.05		0.05	
	Patient 11	0.00		0.02		0.02		0.02		0.02		0.02		0.02		0.02		0.02	
	Patient 22	0.00		0.03		0.03		0.03		0.03		0.03		0.03		0.03		0.03	
	Mean	0.00		0.02		0.03		0.03		0.03		0.03		0.03		0.03		0.03	

^a^Lag numbers correspond to days.

^b^TIB: time in bed.

^c^TST: total sleep time.

## Discussion

In this study, idiographic analyses were applied on time-series data of self-reported sleep and depression ratings in 22 patients with major depressive disorder. Data were collected on a daily basis with a smartphone app across 143-205 days per subject. Significant Granger causal associations were found in 11 of 22 patients regarding the associations between time in bed or total sleep time and depression core symptoms.

The first aim of this study was to determine the number of patients for whom temporal associations could be found to indicate a causal relationship. In 7 patients, temporal associations between time in bed and depression core symptoms were found to exhibit a causal relationship, and causal associations were found between total sleep time and depression core symptoms in 9 patients.

The second aim of the study was to evaluate whether changes in sleep or changes in depression core symptoms are primarily responsible for causing changes in the other variable, and whether more or less sleep causes a reduction in depression core symptoms. The analysis revealed high heterogeneity in these associations among patients. One source of heterogeneity was related to the temporal order of the associations, and hence the direction of a possible causal relationship (Granger causality). Concerning time in bed, the direction of the association was quite equally distributed: time in bed Granger caused depression core symptoms in 3 patients, whereas depression core symptoms Granger caused time in bed in 4 patients. With regard to total sleep time, the association was more homogenous toward total sleep time being the cause rather than the effect: total sleep time Granger caused depression core symptoms in 6 patients, whereas the temporal order was reversed in 3 patients.

A different source of heterogeneity concerns the nature of the relationship (eg, if more sleep or depression core symptoms causes a reduction or an increase in the other variable). The association between time in bed and depression core symptoms was positive in 6 patients, meaning that longer time in bed/more depression core symptoms led to an increase in depression core symptoms/time in bed, whereas this association was negative in 1 patient, meaning that more depression core symptoms led to a decrease in time in bed. A more homogenous pattern was found with regard to the association between total sleep time and depression core symptoms: The association was positive in 5 patients, meaning that longer total sleep time/more depression core symptoms led to more depression core symptoms/longer total sleep time, and the association was negative in 4 patients, meaning that longer total sleep time/depression core symptoms led to a decrease in depression core symptoms/total sleep time.

The third aim was to analyze the impact and temporal dynamics of the effects identified. Variance decomposition revealed that, on average, the largest proportion of explained variance was in the association between time in bed and depression core symptoms, with an average 10% of depression core symptoms being explained by time in bed. The second largest proportion concerned the association between total sleep time and depression core symptoms with 6% of the variance in depression core symptoms being explained by total sleep time. Explained variance was small in associations with a reversed temporal order: 3% of the variance in total sleep time and 3% of the variance in time in bed could be explained by depression core symptoms. Impulse response analysis revealed that changes of 1 SD in a variable (eg, total sleep time) had the greatest impact on the other variable (eg, depression core symptoms) in the following 2 to 4 days.

An advantage of our analysis is that it allows the possibility to make statistical claims on an individual level owing to the use of time series. This would be particularly useful for a clinician since it would be highly relevant to know whether a temporal association between sleep and depression exists in a certain patient and whether it is more likely that sleep changes cause changes in depression or vice versa. If patients are willing to share such data with a clinician [27], it could be used for treatment decisions. For example, if the data suggest that a longer time in bed worsens depressive symptoms, therapeutic sleep restriction or long-term mild sleep restriction might be a promising intervention. This knowledge could also be used by the patients themselves for self-management activities.

## Abbreviations

FEVD: forecast error variance decomposition
IDS-C: Inventory of Depressive Symptomatology Clinician
IRF: impulse response function
PHQ-2: Two-item Health Questionnaire
PSQI: Pittsburg Sleep Quality Index
STEADY: sensor-based system for therapy support and management of depression
VAR: vector autoregression
